# Understanding of black salve toxicity by multi-compound cytotoxicity assays

**DOI:** 10.1186/s12906-022-03721-y

**Published:** 2022-09-20

**Authors:** Andrew Croaker, Arie Davis, Anthony Carroll, Lei Liu, Stephen P. Myers

**Affiliations:** 1 Faculty of Health, Southern Cross University, Lismore, NSW Australia; 2 Toormina Medical Centre Skin Cancer Clinic, Toormina, NSW Australia; 3 School of Pharmacy and Medical Sciences, Griffith University, Gold Coast, Queensland Australia; 4 Griffith Institute for Drug Discovery, Griffith University, Gold Coast, Queensland Australia; 5 Southern Cross Plant Science, Southern Cross University, Lismore, NSW Australia; 6 National Centre for Naturopathic Medicine, Faculty of Health, Southern Cross University, Lismore, NSW Australia; 7 NatMed Research, Evans Head, NSW Australia

**Keywords:** Black salve, Skin cancer, Melanoma, Dermatology, Escharotic

## Abstract

**Background:**

Black salve is a controversial complementary and alternative medicine (CAM) associated with skin toxicity and skin cancer treatment failures. Black salve formulations vary between manufacturers and contain a number of botanical and synthetic constituents. The skin cancer cytotoxicity of a number of these constituents has not been assessed to date. The alkaloids from the rhizomes of *Sanguinaria canadensis*, a key black salve ingredient, have had their single compound cytotoxicity assessed; however, whether they possess synergistic cytotoxicity with other compounds has not been studied and is of direct clinical relevance. This research aimed to improve our understanding of the skin cancer cytotoxicity of black salve constituents.

**Methods:**

The cytotoxicity of individual and combination black salve constituents were assessed against the A375 melanoma and A431 squamous cell carcinoma cell lines. Cytotoxicity was determined using the Resazurin assay with fluorescence measured using a Tecan Infinite 200 Pro Microplate reader, compound cytotoxicity being compared to that of the topical cancer therapeutic agent, 5- fluouracil. Docetaxal was used as a positive control. Dunnetts *p* value was used to determine whether significant synergistic cytotoxicity was present.

**Results:**

Sanguinarine was the most cytotoxic compound tested with a 24-hour IC_50_ of 2.1 μM against the A375 Melanoma cell line and 3.14 μM against the A431 SCC cell line. All black salve constituents showed greater cytotoxicity against the two skin cancer cell lines tested than the skin cancer therapeutic 5-Fluouracil with 24 hours of compound exposure. Chelerythrine and minor Quaternary Benzophenanthridine Alkaloids (QBAs) present in black salve, at concentrations not having a cytotoxic effect by themselves, boosted the cytotoxic effects of sanguinarine. This could be a synergistic rather than additive cytotoxic effect although the synergistic effect was cell line and concentration dependent.

**Conclusions:**

Black salve contains several cytotoxic compounds, a number of which have been found to possess synergistic cytotoxicity for the first time against skin cancer cell lines. In addition, these compounds together increase the overall cytotoxic effect. Assessing multi-compound cytotoxicity in herbal medicine can provide additional information about both their therapeutic and toxicity potential. As black salve is currently being used by patients, further cytotoxicity work should be undertaken to assess whether synergistic cytotoxicity exists when tested in normal skin cells.

**Supplementary Information:**

The online version contains supplementary material available at 10.1186/s12906-022-03721-y.

## Background

In contrast to pharmaceutical medicines that predominantly consist of a single compound designed for a single target, herbal medicines rely on the combined effects of multiple components that may have synergistic or antagonistic interactions [1]. To understand the therapeutic actions of herbal therapies and better determine their safety and potential toxicity profiles, it is therefore important to conduct multicomponent pharmacological testing [2].

*Sanguinaria canadensis* is a key ingredient used in the manufacture of black salve, it has a long history of ethnobotanical use by Native American peoples especially of the Algonquian, Iroquois and Siouan language groups [3, 4]. Traditional uses included as a red skin dye [5], throat lozenge when added to a cube of maple sugar [6], coagulant for axe wounds [7] and as an abortificant [8]. Hearing of its traditional use to treat cancer an American surgeon combined *Sanguinaria canadensis* also known as bloodroot, with zinc chloride creating the first black salve in the 1850s [9].

*Sanguinaria canadensis* contains several alkaloids from the quaternary benzophenanthridine alkaloid (QBA) and protopin groups. While these alkaloids have had their cytotoxic potential assessed as individual compounds [10], the possibility that they possess synergistic cytotoxic potential is currently unknown. This is of direct clinical relevance and may alter regulatory agency guidelines regarding safe and hazardous compounds and herbal extract exposure levels. Safety levels based on single compound cytotoxicity studies may over estimate safe exposure concentrations when in fact synergistic compound cytotoxicity exists within the same herbal medicine [11].

Black salves represent a heterogenous product group that have significant compositional and constituent concentration variability between manufacturers [12]. In addition to *S. canadensis*, black salves contain high concentrations of zinc chloride, with one black salve manufacturers product also containing *Larrea mexicana* (chapparal leaf) with 17% nordihydrogauaretic acid (NDGA) by weight. Assessing the cytotoxic potential of these additional constituents will provide a more comprehensive overview of black salves’ potential as a cancer therapy and its toxicity risks.

Sanguinarine, the main alkaloid by weight present in black salve, has been used to gauge toxin exposure in patients suffering from the condition epidemic dropsy with a correlation found between serum sanguinarine levels and the severity of epidemic dropsy symptoms [13]. Whether sanguinarine black salve concentrations could similarly be used to assess the risk of black salve toxicity in skin lesions has not been determined. To properly assess the cytotoxic potential of black salve formulations the cytotoxicity we assessed the cytotoxicity of the *S. canadensis* alkaloid pool containing six quarternary benzophenanthridine alkaloids (sanguinarine; chelerythrine; sanguilutine; chelilutine; sanguirubine and chelirubine) and two protopin alkaloids (protopine and allocryptopine).

## Methods

### Experiment design and research work

Cytotoxicity assay work was undertaken at Griffith University School of Pharmacy and Pharmacology laboratory, Gold Coast, Queensland. The work being conducted by research assistant Arie Davis. Alkaloids were isolated from *S. canadensis* rhizomes, purified and validated at the Griffith Research Institute for Drug Discovery by Professor Anthony Carroll and research assistant Darren Holland. Single compound and combination compound cytotoxicity experiments were designed by Andrew Croaker with guidance by Assoc. Prof Shailendra Anoopkumar-Dukie from the Griffith University School of Pharmacy and Pharmacology.

### Bloodroot rhizomes

*S. canadensis* rhizomes were purchased from Pacific Botanicals (Oregon, United States). A voucher specimen of the rhizomes has been retained in the Medicinal Plant Herbarium, herbarium voucher number: PHARM-18-0078, Southern Cross University, Australia. The rhizomes were confirmed as *S. canadensis* by Mr. Peter Mouatt after comparison with herbarium sample voucher number: PHARN-12-0480 at Southern Cross University.

### Nuclear magnetic resonance (NMR) procedures

NMR spectra were acquired at 25 °C using a Bruker Biospin GmbH 500 MHz spectrometer (Massachusetts, US) equipped with a triple (TCl) resonance 5 mm probe, 2D NMR spectra were acquired using standard Bruker pulse sequences. The solvent used during NMR analysis was Cambridge isotopes DMSO*-d*_6_ (D, 99.9%) or CDCl3 (D, 99.9%) (Massachusetts, US). Spectra were referenced to solvent peaks at *δ*_H_ 2.50 (^1^H) and *δ*_C_ 39.52 (^13^C) for DMSO-*d*_6_ or *δ*_H_ 7.26 (^1^H) and *δ*_C_ 77.0 (^13^C) for CDCl_3_.

### High performance liquid chromatography (HPLC)/ mass spectroscopy (MS) instruments, columns and reagents

An Agilent 5530 Accurate Mass Quadrupole Time Of Flight (QTOF) Liquid Chromatography (LC)/ MS (CA, US) was used to obtain high resolution (+) Electrospray Ionization (ESI) MS data. Alltech Davisil 30–40 um 60 Å C_18_ bonded silica gel (Grace Davison Discovery Sciences, MD, US) was used to adsorb the sample prior to HPLC separation and for Medium Pressure Liquid Chromatography (MPLC) separations. A Merck Hitachi L7100 pump equipped with a Merck Hitachi L7455 Photodiode Array (PDA) detector (Hitachi Ltd., Tokyo, Japan) were also used for HPLC analysis. HPLC columns used include a Thermo Betasil C_18_ 5 μm, 100 Å, 150 mm × 21.2 mm, (Thermofisher Scientific, Massachusetts, US) and a Thermo Betasil C_18_ 5 μm, 100 Å, 250 mm × 10 mm and Phenyl bonded silica HPLC column 5 μm, 100 Å, 250 mm × 21.2 mm (Thermofisher Scientific, Massachusetts, US). Ion exchange chromatography was done using Amberjet 1200H strong acid cation exchanger (Rohm and Haas, Philadelphia, US). The fractions from the HPLC separation were dried using a GeneVac HT-12 centrifugal evaporator (SP Scientific, NY, USA). All solvents used during HPLC and MS were HPLC grade. All solvents used for HPLC and MS were Lab-Scan HPLC grade (RCI Labscan, Bangkok, Thailand), the H_2_O being Millipore Milli-Q PF filtered. Trifluoroacetic acid (TFA) was spectroscopy grade from Alfa Aesar (Thermofisher Scientific, NY, US).

### *S. canadensis* alkaloid extraction and isolation

The dried ground sample of *S. canadensis* (167 g) was exhaustively extracted using MeOH (4 × 300 mL) to yield a deep red extract. This extract was filtered through Amberjet 1200H strongly acidic cation exchange resin (100 g). The resin was then washed with MeOH (200 mL) and the alkaloids eluted from the resin with ammonia solution (28%, 100 mL) followed by MeOH (300 mL). These fractions were combined and then dried to yield a dark red gum (5.5 g). This material was adsorbed on to C_18_ silica gel at a 1:1 ratio and packed into the top of a bed of preconditioned C_18_ silica gel (200 g) in a MPLC (MPLC) column (40 mm × 150 mm). The columns were eluted with a gradient from 99.9% H_2_O containing 0.1% TFA to 99.9% MeOH containing 0.1% TFA over 120 min at a flow rate of 9 mL/min. The column was further eluted with 99.9% MeOH containing 0.1% TFA for 30 min. A total of 150 fractions were collected at 1 min intervals. Fractions were analysed by (+) ESI MS and fractions containing the same molecular ions were combined and dried. This resulted in semi-pure fractions containing mixtures of *m/z* 354/370, *m/z* 332/348 and *m/z* 362/378/394. These semi-pure fractions were then repeatedly purified by Reversed Phase (RP) HPLC on either Betasil C_18_ bonded silica preparative HPLC column (21 mm × 150 mm) (Thermofisher Scientific, Massachusetts, US) or Phenyl bonded silica preparative HPLC column (21 mm × 250 mm) (Thermofisher Scientific, Massachusetts, US) column eluted with a gradient from 50% H_2_O/50% MeOH to 100% MeOH over 60 min at a flow rate of 9 mL/min. The column was then further eluted with 100% MeOH for 10 min. This resulted in sanguinarine (36.5 mg), chelerythrine (33.4 mg), sanguilutine (9.1 mg), chelilutine (7.8 mg), sanguirubine (1.2 mg), chelirubine (2.5 mg), protopine (4.1 mg) and allocrytopine (4.2 mg) being purified. All compounds were characterised by 1H NMR spectroscopy to confirm their structures .

#### Compound stock solutions

##### *S. canadensis* alkaloids

Master Stock Solutions of individual alkaloids were made to 10 mM in methanol from which working stock solutions were made. All alkaloids were stored protected from light at − 20 °C. Working stock concentrations were diluted in culture media and were 0.5 μM to 5 μM for sanguinarine; 3.25 μM to 50 μM for chelerythrine; 5 μM to 50 μM for sanguilutine; 5 μM to 25 μM for chelilutine and 10 μM to 20 μM for chelirubine; 3.125 μM to 100 μM for protopine and 50 μM to 150 μM for allocryptopine.

**Fig. 1:**
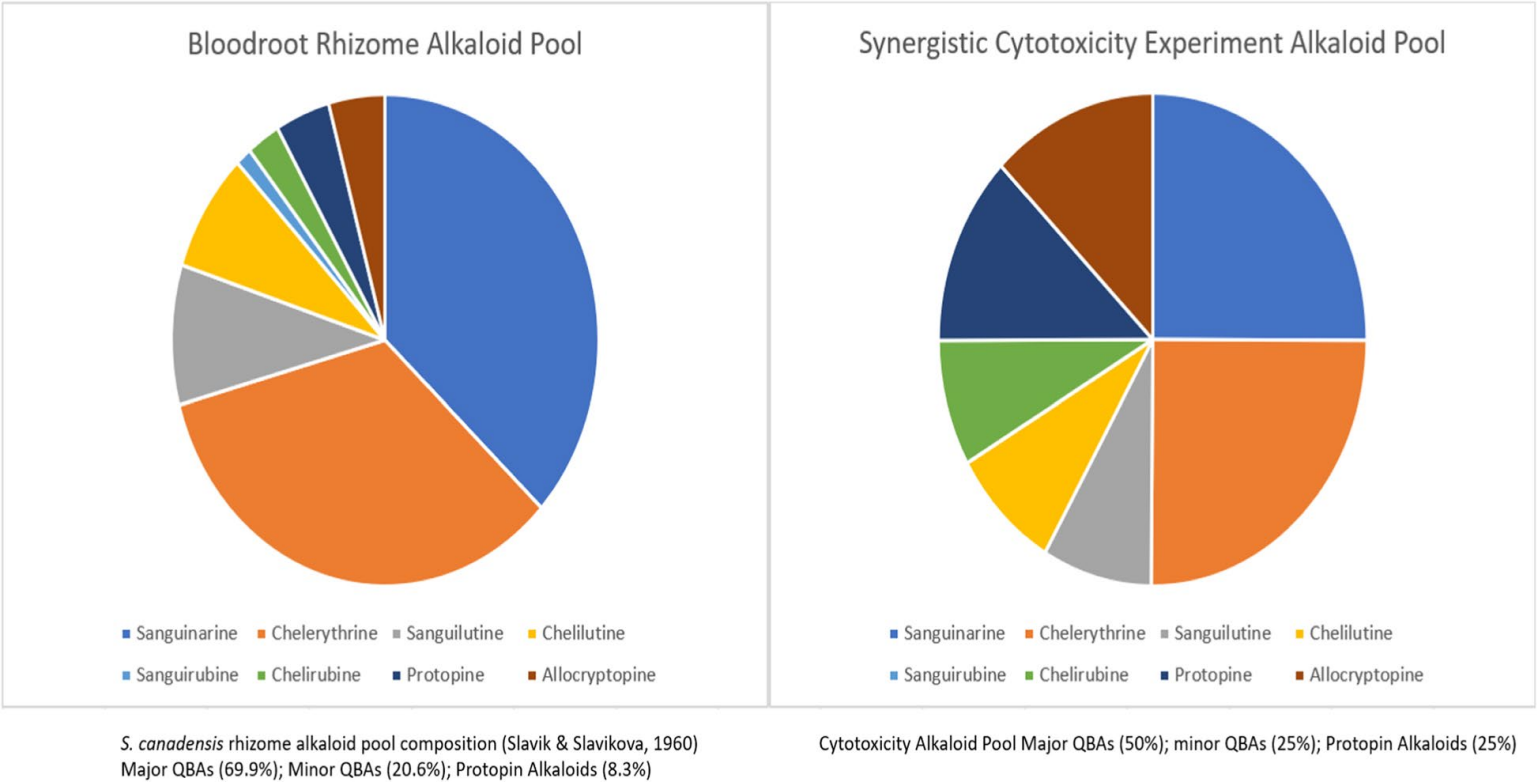
Comparison of bloodroot rhizome and combination cytotoxicity experiment alkaloid pools

For combination alkaloid cytotoxicity studies, an alkaloid mixture to reflect the alkaloid pool found in *S. canadensis* rhizomes was used. The alkaloids present in *S. canadensis* rhizomes show significant natural variability as previously reviewed [14]. A comparison of the bloodroot alkaloid mix used in our synergistic cytotoxicity experiments compared to the *S. canadensis* alkaloid rhizome pool first reported by [15] is shown in Fig. 1.

Total alkaloid pool combination cytotoxicity experiments used the following concentration ratio.

**Table:** 

Sanguinarine	Chelerythrine	Sanguilutine	Chelilutine	Chelirubine	Protopine	Allocryptopine
1	1	1/3	1/3	1/3	1/2	1/2

The 1 μM total alkaloid combination for example contained 1 μM sanguinarine, 1 μM chelerythrine 0.33 μM sanguilutine, 0.33 μM chelilutine and 0.33 μM chelirubine, 0.5 μM allocryptopine and 0.5 μM protopine. The 2 μM total alkaloid combination contained sanguinarine 2 μM; chelerythrine 2 μM; Minor QBAs 2 μM (Sanguilutine 0.66 μM/ Chelilutine 0.66 μM/ Chelirubine 0.66 μM); Protopin 2 μM (Protopine 1 μM/ Allocryptopine 1 μM).

##### Noridihydroguaiaretic acid (NDGA)

NDGA found in *Larrea mexicana* (chapparal leaf) was purchased from Sigma Aldrich (MO, USA). Stock solutions of NDGA were made to 10 mM in methanol. Working solutions from 100 μM to 200 μM were diluted in culture media and used throughout 24 h experiments. Stock solutions were protected from light and stored at − 20 °C.

##### Zinc chloride (ZnCl_2_)

Zinc Chloride used as a major component of black salve preparations was purchased from Sigma Aldrich (MO, USA). Stock solutions of ZnCl_2_ were made to 10 mM in methanol. Working solutions from 100 μM to 500 μM were diluted in culture media and used throughout 24 h experiments. Stock solutions were protected from light and stored at − 20 °C.

##### 5-FU

5-FU was purchased from Sigma Aldrich (MO, USA) with stock solutions made to 20 mM in PBS. For 24 h experiments, working solutions from 1 mM to 1.5 mM were prepared and diluted in culture media. For 72 h experiments, solutions ranging from 2.5 μM to 30 μM were prepared and diluted in culture media. Stock solutions were protected from light and stored at − 20 °C. 5-FU was included as an active control.

### Cell culture

Malignant melanoma A375 and squamous cell carcinoma A431 cell lines were purchased from American Type Culture Collection (ATCC) (VA, USA). Cells were cultured in Falcon 75cm^3^ flasks to 90% confluency, and kept in a humidified incubator at 37 °C with 5% CO_2_. Cells were cultured in Dulbecco’s Modified Eagle Medium (DMEM), 4.5 g/L glucose, sodium pyruvate, L-Glutamine, and Phenol RedMedium (Gibco, Victoria, Australia) were supplemented with 10% foetal bovine serum (FBS) (Scientifix, Victoria, Australia) and 0.1 mg/mL gentamicin (Gibco, Victoria, Australia).

### Alamar blue (Resazurin) assay

A375 cells were seeded at 1.5 × 10^4^ cells per well, and A431 cells were seeded at 1 × 10^4^ cells per well in 96-well plates. Plates were incubated at 37 °C with 5% CO_2_. At 24 h, cells were treated with chosen black salve constituents at a range of single compound and multi-compound concentrations. Respective solvent was used as the vehicle control in all experiments. At 24 h, 48 h or 72 h, the media above cells was removed and replaced with 200 μL of media containing 44 μM Resazurin solution (Sigma, MO, USA) and incubated for 3 h at 37 °C with 5% CO_2_. At 3 h, reduction of resazurin to resorufin was measured using a Tecan Infinite 200 Pro Microplate reader (excitation: 530 nm, emission: 590 nm) (Tecan, NSW, Australia). Cells without treatment compounds incubated for 24 h, 48 h or 72 h were used to determine 100% viability. Cell free controls of only rezasurin containing-media with and without test compounds were used to determine background fluorescence levels due to the autofluorescence of some target compounds. Docetaxel 100 μM (Sapphire Bioscience, NSW, Australia) was used as a cytotoxicity positive control.

### Analysis of cytotoxicity results

Cell viability was determined by measuring the fluorescence as a % of solvent control. Concentration viability response curves, IC_50_ values, mean IC_50_ value and standard error were generated using Graphpad Prism 8 for Windows (Graphpad Software, La Jolla, CA, USA, www.graphpad.com). Each experiment was repeated in triplicate on each plate, with each experiment also repeated on at least three different days.

## Results

### Individual compound cytotoxicity

Our analysis revealed there are multiple constituents in black salve that possess cytotoxicity against two different human skin cancer cell lines (Table 1). Sanguinarine was the most cytotoxic compound tested with a 24-hour IC50 of 2.1 μM against the A375 Melanoma cell line and 3.14 μM against the A431 SCC cell line. Chelerythrine the other major QBA, was the next most cytotoxic compound with a 24-hour IC50 of 10.29 μM against the A375 cell line and 10.79 μM against the A431 cell line. The minor QBAs also showed significant individual compound cytotoxicity against both cell lines with chelirubine being the most cytotoxic of the three minor QBAs tested having a 24-hour IC_50_ of 11.88 μM against the A375 cell line and 14.43 μM against the A431 cell line.

**Table 1:** Black Salve constituent 24 hour cytotoxicity analysis

Cell Line	A375 Melanoma		A431 SCC	
Compound	IC50 (μM)	S.E.	IC50 (μM)	S.E.
Sanguinarine	2.1	0.15	3.14	0.3
Chelerythrine	10.29	0.71	10.79	0.5
Sanguilutine	25.7	2.98	15.34	2.73
Chelilutine	16.15	0.33	16.5	1.04
Chelirubine	11.88	1.09	14.43	0.46
Protopine	> 200		> 200	
Allocryptopine	312.67	38.58	276.96	12.37
NDGA	117.94	11.3	201.96	7.52
ZnCl_2_	334.74	12.28	612.35	15.37
5-FU	1046.43	77.08	> 2000	

Positive Control Docetaxel 100 μM A375 24 hr. viability 8.78% A431 24 hr. viability 29.65%

IC50 cytotoxicity results with Alamar Blue Emission Spectra

The protopin alkaloids were found to be much less cytotoxic than the major and minor QBAs. Allocryptopine was approximately 100 times less cytotoxic than sanguinarine and approximately 10 to 30 times less cytotoxic than the minor QBAs. Further the IC_50_ of protopine was not reached when testing at concentrations up to 200 μM. Previous analysis of black salve alkaloid constituent concentrations suggests the majority of black salves contain individual alkaloid concentrations several orders of magnitude higher than that required to cause cytotoxicity against malignant and non-malignant human skin cells [12].

ICP-MS analysis of a number of black salve products has found they contain between 20 to 45% zinc chloride by weight, easily exceeding the zinc chloride IC_50_ concentrations of 334.74 μM for the A375 and 612.35 μM for the A431 cell lines [12]. One manufacturer also lists chapparal leaves containing 17% NDGA as the second highest black salve ingredient by weight in their formulation behind zinc chloride (Alpha Omegalabs https://www.alphaomegalabs.com/cansemar-black-topical-salve-22g.html). Despite the IC_50_ values of NDGA being higher than the majority of the alkaloids present in black salve at 117.94 μM against the A375 and 201.96 μM against the A431 cell lines, it is expected they are present in sufficiently high concentrations in black salve to exceed their IC_50_ values. This suggests that apart from the alkaloid constituents of black salve, non-alkaloid constituents would also be expected to contribute significantly to its cytotoxic effect and are likely to also pose a risk to normal tissues**.**

Of interest, all black salve constituents showed greater cytotoxicity against the two skin cancer cell lines tested than the skin cancer therapeutic 5-Fluouracil with 24 hours of compound exposure. The cytotoxicity of 5-FU was considerably increased against the A375 cell line after 72 hours of compound exposure with an IC_50_ of 10.81 μM while sanguinarine still had a superior cytotoxic effect with an IC_50_ value of 1.77 μM at 72 hours (similar to that at 24 hrs).

### Combination compound cytotoxicity

**Fig. 2:**
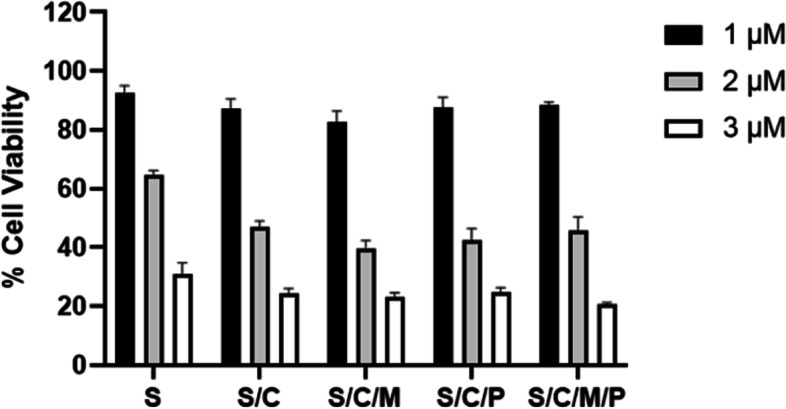
24Hr A375 bloodroot combination alkaloid cytotoxicity. S = Sanguinarine Alkaloid 1 μM; 2 μM or 3 μM. S/C = Sanguinarine and Chelerythrine Alkaloid Combination; 1 μM/1 μM; 2 μM/2 μM; 3 μM/3 μM. S/C/M = Sanguinarine and Chelerythrine and Minor QBAs Combination; 1 μM Group: Sanguinarine 1 μM; Chelerythrine 1 μM; Minor QBA 1 μM (Sanguilutine 0.33 μM/ Chelilutine 0.33 μM/ Chelirubine 0.33 μM). 2 μM Group: Sanguinarine 2 μM; Chelerythrine 2 μM; Minor QBA (Sanguilutine 0.66 μM/ Chelilutine 0.66 μM/ Chelirubine 0.66 μM). 3 μM Group: Sanguinarine 3 μM; Chelerythrine 3 μM; Minor QBA (Sanguilutine 1 μM/ Chelilutine 1 μM/ Chelirubine 1 μM). S/C/M/P = Sanguinarine and Chelerythrine and Minor QBAs and Protopin Alkaloid Combination. 1 μM Group: Sanguinarine 1 μM; Chelerythrine 1 μM; Minor QBA 1 μM (Sanguilutine 0.33 μM/ Chelilutine 0.33 μM/ Chelirubine 0.33 μM); Protopin 1 μM (Protopine 0.5 μM/ Allocryptopine 0.5 μM). 2 μM Group: Sanguinarine 2 μM; Chelerythrine 2 μM; Minor QBA (Sanguilutine 0.66 μM/ Chelilutine 0.66 μM/ Chelirubine 0.66 μM); Protopin 2 μM (Protopine 1 μM/ Allocryptopine 1 μM). 3 μM Group: Sanguinarine 3 μM; Chelerythrine 3 μM; Minor QBA (Sanguilutine 1 μM/ Chelilutine 1 μM/ Chelirubine 1 μM); Protopin 3 μM (Protopine 1.5 μM/ Allocryptopine 1.5 μM)

In addition to the presence of multiple cytotoxic compounds in black salve formulations that exert their own individual cytotoxic effects, these compounds may work synergistically or antagonistically resulting in the black salve alkaloid pool being more or less cytotoxic than the sum of its parts. To explore this possibility, we compared the individual to the combined cytotoxicity of black salve alkaloids. The data presented in Fig. 2, shows the cytotoxicity of *S. canadensis* alkaloid combinations to that of sanguinarine alone against the A375 melanoma cell line. To determine whether chelerythrine, the minor QBA alkaloid or protopin alkaloid groups possessed synergistic or antagonistic cytotoxicity effects, they were added separately and in combination to sanguinarine.

**Fig. 3:**
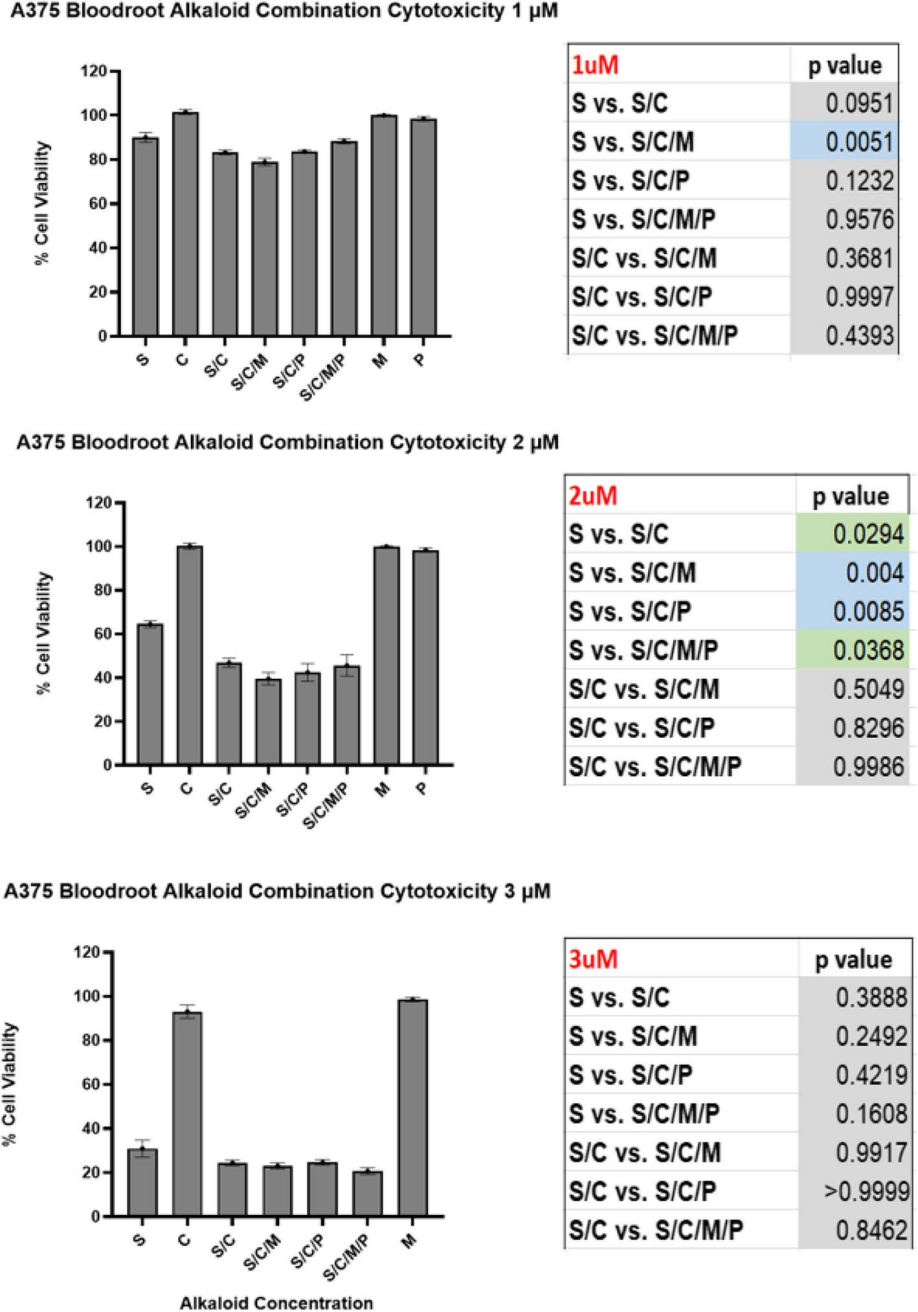
Statistically significant A375 synergistic cytotoxicity. S = Sanguinarine Alkaloid 1 μM; 2 μM or 3 μM. C = Chelerythrine Alkaloid 1 μM; 2 μM or 3 μM. S/C = Sanguinarine and Chelerythrine Alkaloid Combination; 1 μM/1 μM; 2 μM/2 μM; 3 μM/3 μM. S/C/M = Sanguinarine and Chelerythrine and Minor QBAs Combination; 1 μM Group: Sanguinarine 1 μM; Chelerythrine 1 μM; Minor QBA 1 μM (Sanguilutine 0.33 μM/ Chelilutine 0.33 μM/ Chelirubine 0.33 μM). 2 μM Group: Sanguinarine 2 μM; Chelerythrine 2 μM; Minor QBA (Sanguilutine 0.66 μM/ Chelilutine 0.66 μM/ Chelirubine 0.66 μM). 3 μM Group: Sanguinarine 3 μM; Chelerythrine 3 μM; Minor QBA (Sanguilutine 1 μM/ Chelilutine 1 μM/ Chelirubine 1 μM). S/C/M/P = Sanguinarine and Chelerythrine and Minor QBAs and Protopin Alkaloid Combination. 1 μM Group: Sanguinarine 1 μM; Chelerythrine 1 μM; Minor QBA 1 μM (Sanguilutine 0.33 μM/ Chelilutine 0.33 μM/ Chelirubine 0.33 μM); Protopin 1 μM (Protopine 0.5 μM/ Allocryptopine 0.5 μM). 2 μM Group: Sanguinarine 2 μM; Chelerythrine 2 μM; Minor QBA (Sanguilutine 0.66 μM/ Chelilutine 0.66 μM/ Chelirubine 0.66 μM); Protopin 2 μM (Protopine 1 μM/ Allocryptopine 1 μM). 3 μM Group: Sanguinarine 3 μM; Chelerythrine 3 μM; Minor QBA (Sanguilutine 1 μM/ Chelilutine 1 μM/ Chelirubine 1 μM); Protopin 3 μM (Protopine 1.5 μM/ Allocryptopine 1.5 μM). M = Minor QBA Combination;1 μM Group: Sanguilutine 0.33 μM/ Chelilutine 0.33 μM/ Chelirubine 0.33 μM. 2 μM Group: Sanguilutine 0.66 μM/ Chelilutine 0.66 μM/ Chelirubine 0.66 μM. 3 μM Group: Sanguilutine 1 μM/ Chelilutine 1 μM/ Chelirubine 1 μM. Cell viability percentage compared to controls; Cell viability assessed after 24 hr. compound exposure; Cell viability standard error bars; Significance calculated using Dunnetts *p* value method

At concentrations of 1 and 2 μM chelerythrine, the minor QBAs and protopin alkaloids as individual agents lacked a cytotoxic effect against the A375 cell line. This being the case, any statistically significant increase in cytotoxicity compared to sanguinarine alone at these concentrations would indicate a synergistic cytotoxic effect. As can be seen in Fig. 3, a synergistic cytotoxic effect for a combination of chelerythrine and the minor QBAs was found at a concentration of 1 μM (*p* = 0.005), while at 2 μM a range of alkaloid combinations showed synergistic cytotoxicity (*p* values 0.004 to 0.037). Compared to 2 μM of sanguinarine alone, the addition of chelerythrine and the minor QBAs reduced A375 viability from 64.5 to 39.6%, this being a 38.6% increase in cytotoxicity (*p* = 0.004).

**Fig. 4:**
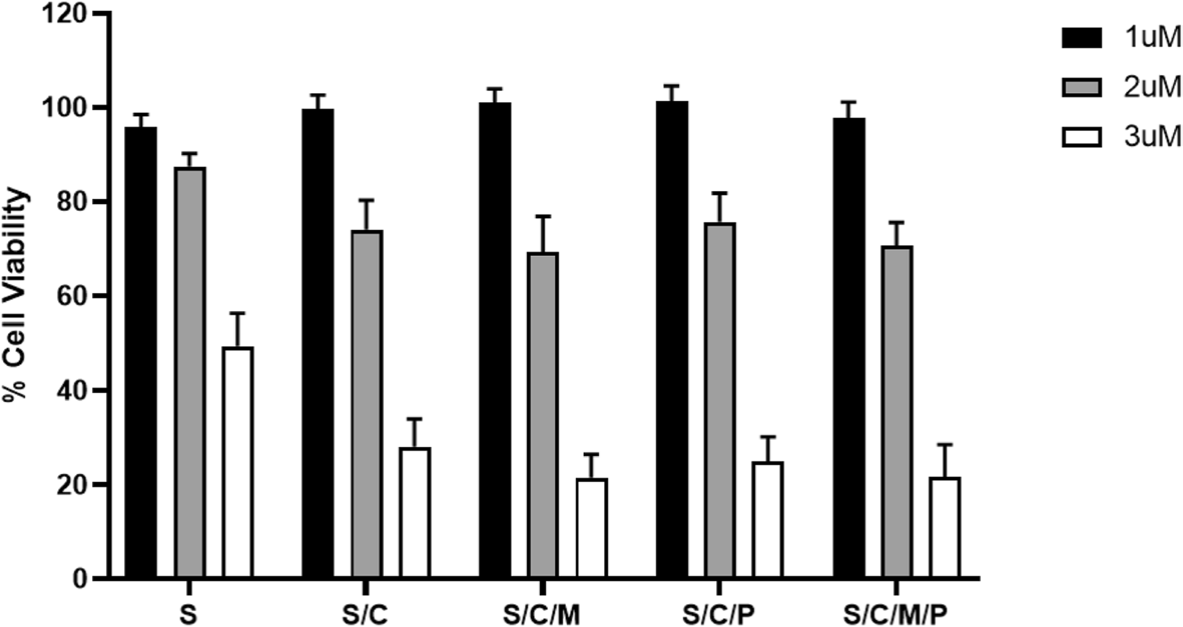
24Hr A431 bloodroot combination alkaloid cytotoxicity. S = Sanguinarine Alkaloid 1 μM; 2 μM or 3 μM. S/C = Sanguinarine and Chelerythrine Alkaloid Combination; 1 μM/1 μM; 2 μM/2 μM; 3 μM/3 μM. S/C/M = Sanguinarine and Chelerythrine and Minor QBAs Combination; 1 μM Group: Sanguinarine 1 μM; Chelerythrine 1 μM; Minor QBA 1 μM (Sanguilutine 0.33 μM/ Chelilutine 0.33 μM/ Chelirubine 0.33 μM). 2 μM Group: Sanguinarine 2 μM; Chelerythrine 2 μM; Minor QBA (Sanguilutine 0.66 μM/ Chelilutine 0.66 μM/ Chelirubine 0.66 μM). 3 μM Group: Sanguinarine 3 μM; Chelerythrine 3 μM; Minor QBA (Sanguilutine 1 μM/ Chelilutine 1 μM/ Chelirubine 1 μM). S/C/M/P = Sanguinarine and Chelerythrine and Minor QBAs and Protopin Alkaloid Combination. 1 μM Group: Sanguinarine 1 μM; Chelerythrine 1 μM; Minor QBA 1 μM (Sanguilutine 0.33 μM/ Chelilutine 0.33 μM/ Chelirubine 0.33 μM); Protopin 1 μM (Protopine 0.5 μM/ Allocryptopine 0.5 μM). 2 μM Group: Sanguinarine 2 μM; Chelerythrine 2 μM; Minor QBA (Sanguilutine 0.66 μM/ Chelilutine 0.66 μM/ Chelirubine 0.66 μM); Protopin 2 μM (Protopine 1 μM/ Allocryptopine 1 μM). 3 μM Group: Sanguinarine 3 μM; Chelerythrine 3 μM; Minor QBA (Sanguilutine 1 μM/ Chelilutine 1 μM/ Chelirubine 1 μM); Protopin 3 μM (Protopine 1.5 μM/ Allocryptopine 1.5 μM)

**Fig. 5:**
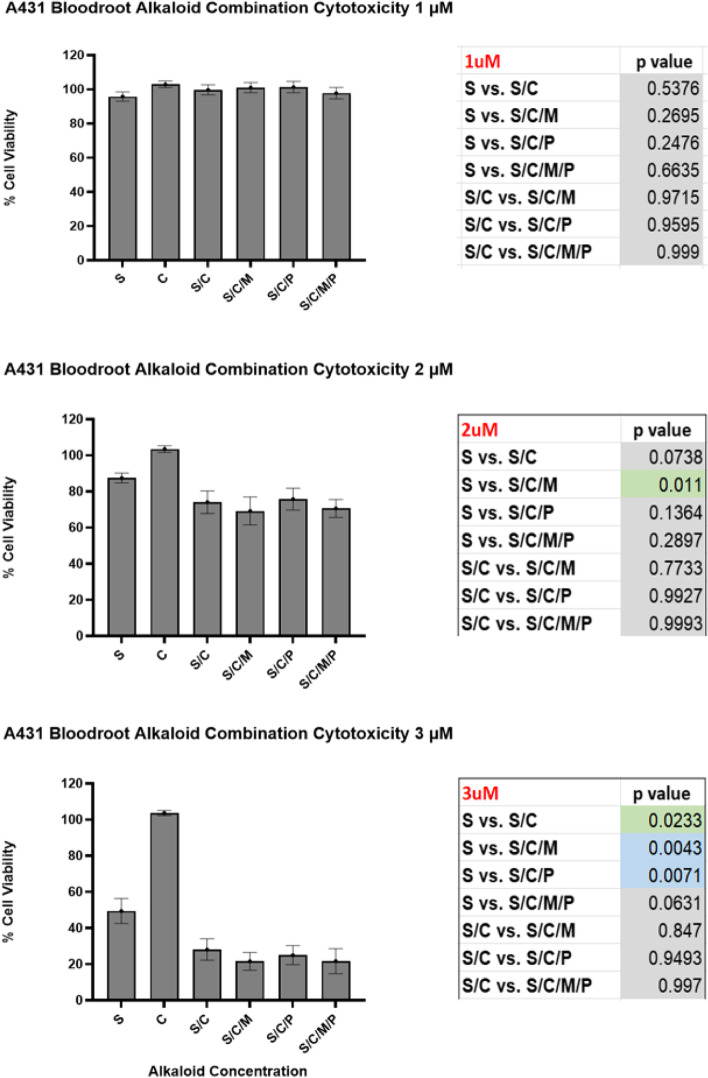
Statistically significant A431 synergistic cytotoxicity. S = Sanguinarine Alkaloid 1 μM; 2 μM or 3 μM. C = Chelerythrine Alkaloid 1 μM; 2 μM or 3 μM. S/C = Sanguinarine and Chelerythrine Alkaloid Combination; 1 μM/1 μM; 2 μM/2 μM; 3 μM/3 μM. S/C/M = Sanguinarine and Chelerythrine and Minor QBAs Combination; 1 μM Group: Sanguinarine 1 μM; Chelerythrine 1 μM; Minor QBA 1 μM (Sanguilutine 0.33 μM/ Chelilutine 0.33 μM/ Chelirubine 0.33 μM). 2 μM Group: Sanguinarine 2 μM; Chelerythrine 2 μM; Minor QBA (Sanguilutine 0.66 μM/ Chelilutine 0.66 μM/ Chelirubine 0.66 μM). 3 μM Group: Sanguinarine 3 μM; Chelerythrine 3 μM; Minor QBA (Sanguilutine 1 μM/ Chelilutine 1 μM/ Chelirubine 1 μM). S/C/M/P = Sanguinarine and Chelerythrine and Minor QBAs and Protopin Alkaloid Combination. 1 μM Group: Sanguinarine 1 μM; Chelerythrine 1 μM; Minor QBA 1 μM (Sanguilutine 0.33 μM/ Chelilutine 0.33 μM/ Chelirubine 0.33 μM); Protopin 1 μM (Protopine 0.5 μM/ Allocryptopine 0.5 μM). 2 μM Group: Sanguinarine 2 μM; Chelerythrine 2 μM; Minor QBA (Sanguilutine 0.66 μM/ Chelilutine 0.66 μM/ Chelirubine 0.66 μM); Protopin 2 μM (Protopine 1 μM/ Allocryptopine 1 μM). 3 μM Group: Sanguinarine 3 μM; Chelerythrine 3 μM; Minor QBA (Sanguilutine 1 μM/ Chelilutine 1 μM/ Chelirubine 1 μM); Protopin 3 μM (Protopine 1.5 μM/ Allocryptopine 1.5 μM). Cell viability percentage compared to controls; Cell viability assessed after 24 hr. compound exposure; Cell viability standard error bars; Significance calculated using Dunnetts *p* value method

Against the A431 SCC cell line bloodroot alkaloids showed significant 24-hour cytotoxicity at low concentrations (Fig. 4). Significant synergistic cytotoxicity was also found (Fig. 5) with A431 viability reducing from 49.4% with single compound 3 μM sanguinarine to 21.5% with the addition of chelerythrine and Minor QBAs at a 3 μM concentration where they lacked individual cytotoxicity (*p* = 0.004). This suggested the cytotoxic effect of bloodroot alkaloids to be synergistic and not simply additive.

## Discussion and conclusion

To date, the primary methods used to assess in vitro cell viability and test the cytotoxicity of black salve constituents have been problematic. An analysis of the QBA literature shows that the MTT assay has been the major assay employed to assess QBA compound cytotoxic effects [16]. MTT (3-(4,5-dimethylthiazol-2-yl)-2,5-diphenyl tetrazolium bromide), a yellow tetrazole salt, is reduced in the inner mitochondrial membrane to a purple formazan salt by the action of succinate dehydrogenase. The generation of formazan indicates mitochondrial activity reflecting viable cells [17]. The dye is then extracted from cells and quantified by spectrophotometry [18]. The MTT assay is a rapid colorimetric quantitative method for initial-stage in vitro drug screening [19].

Tetrazolium-based assays can be influenced by chemicals exhibiting redox capacity such as plant extracts [20], polyphenols [21], vitamins [22], and some metallic compounds and alloys [23]. This raises concerns regarding the accuracy of reported QBA alkaloid IC_50_ values as the alkaloids present in *S. canadensis* possess oxidant and anti-oxidant effects [14] that may alter MTTs redox state [20, 24] potentially interfering with assay results. The MTT assay for example when used to assess the cytotoxicity of the green tea polyphenol (−)- epigallocatechin-3-gallate (EGCG) was found to produce a two-fold underestimation of its antiproliferative effects against LNCaP and MCF-7 cells compared to ATP or DNA based assays [21].

Cell lines with a high basal reduction capacity may render cytotoxicity results obtained from the MTT assay unreliable [25]. Epidermal keratinocytes contain antioxidant levels 200% higher than those found in fibroblasts [26] suggesting their use with the MTT assay may generate misleading results [27]. For the reasons listed above the MTT assay was not selected for assessing black salve alkaloid cytotoxicity.

Unlike the tetrazolium chemistries that measure reductive capacity by colorimetric means [28], the product of resazurin reduction (resorufin) can also be measured using fluorescence with a fluorometer equipped with a 560 nm excitation and 590 nm emission filter set. When in its oxidized form, the dye is dark blue in colour and possesses little intrinsic fluorescence. When reduced by the metabolic activity of mitochondrial enzymes in living cells it becomes the pink and highly fluorescent compound resazurin [29]. Nonviable cells rapidly lose their metabolic capacity, do not reduce the dye and thus do not generate a fluorescent signal. The rezasurin assay when used following the manufacturers 3 hour incubation protocol does not have a cytotoxic effect on the cell lines being studied [30]. Resazurin cytotoxicity assays have been found to have a high sensitivity detecting as few as 80 live cells in a well [31].

The fluorescence signal generated by rezasurin reduction assays depends upon the cell types used, the cells metabolic rate and plating density. Z^1^ factor scores of 0.8 to 0.87 for cell densities of 500 to 2500 cells/ well respectively suggest it is an excellent assay for high throughput compound screening [32]. While resazurin assays are cost-effective [33], they are subject to interference by compounds that have redox effects and compounds with intrinsic fluorescence characteristics [34–36].

**Fig. 6:**
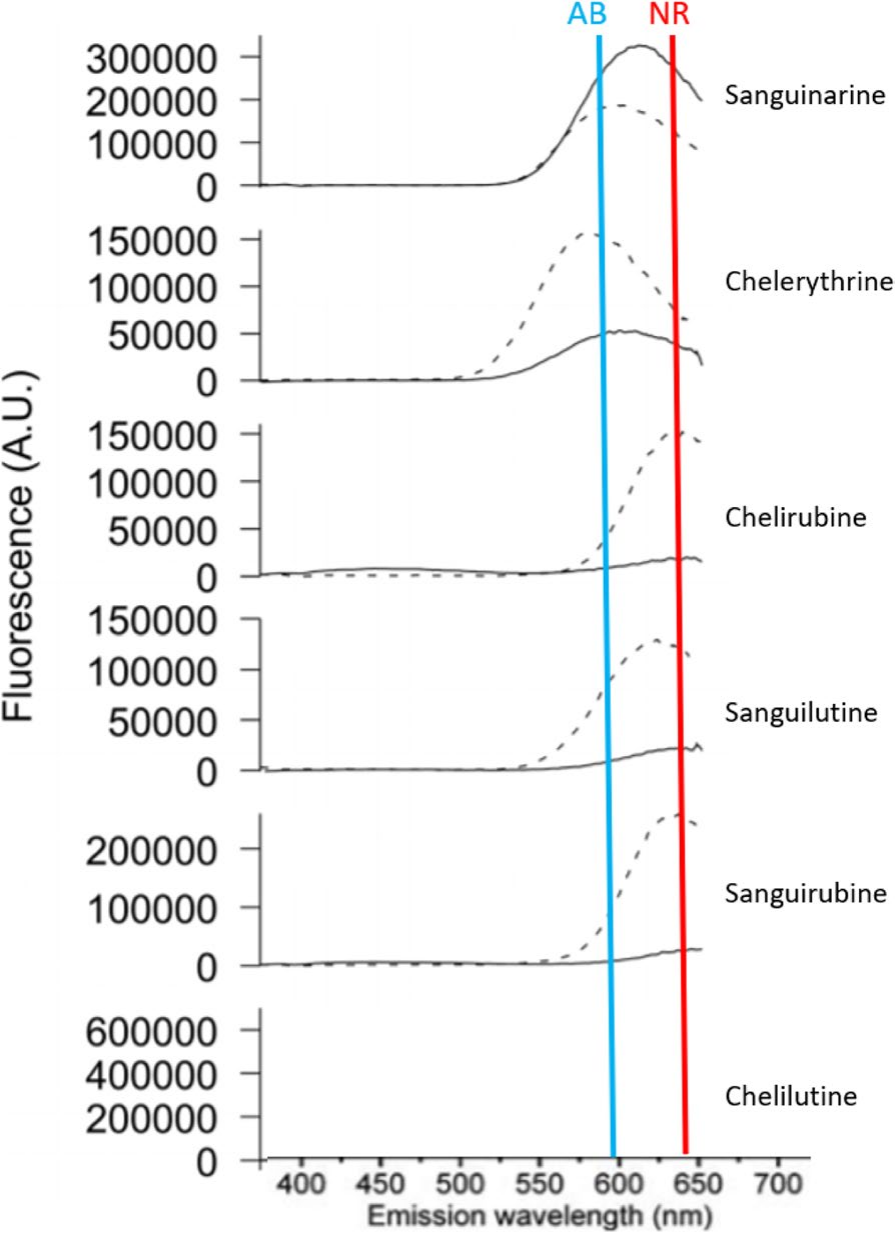
QBA autofluorescence emission wavelengths in the presence or absence of DNA and the detection wavelengths of alamar blue and neutral red cytotoxicity assays. Adapted from [40]. - presence of DNA ^____^ absence of DNA. AB Alamar Blue Emission Spectra 590 nm NR Neutral Red Emission Spectra 645 nm

The QBAs of *S. canadensis* are fluorescent compounds that have been assessed for their suitability as fluorescent DNA probes [37]. Having significant intrinsic fluorescence they may generate interference for fluorescence based cytotoxicity assays. This interference potential has already been demonstrated by sanguilutine and chelilutine causing propidium iodine fluorescence interference [38]. The absorption and emission spectra of some of these alkaloids such as chelirubine, changes significantly upon exposure to DNA [39]. Control wells lacking DNA may therefore not reveal alkaloid interference due to spectral shifts. The reported emission spectra of the individual alkaloids when cultured with or without DNA is shown below (Fig. 6). The fluorescent emission wavelength of the two cytotoxicity assays Alamar Blue and Neutral Red are also shown on this diagram. This suggests Alamar Blue is likely to experience less autofluorescence interference during cytotoxicity assay testing, perhaps with the exception of the alkaloid chelerythrine. The reduced interference is especially important when investigating the cytotoxicity of combinations of autofluorescent alkaloids with various overlapping emission spectra.

Analysis of High Throughput Screening (HTS) compound libraries have found that approximately 2–5% of compounds fluoresce in the blue spectral region λ 350 nm / λ 440 nm wheres only 0.004–0.01% of compounds fluoresce in the λ 560 nm / λ 585 nm red spectral region typical of resorufin [41, 42]. The alkaloids of *S. canadensis* have absorption and emission spectra that includes the longer wavelength red spectrum.

Despite black salve escharotics being associated with local toxicities and treatment failures for several decades [40], the cytotoxic potential of several of its constituents and whether they possess synergistic cytotoxic effects has not previously been studied. This initial assessment suggests that multiple constituents at concentrations found in black salve formulations will have significant cytotoxicity. Several of these constituents have been shown to lack cancer specificity, possessing destructive activity against both malignant and non-malignant human skin cells [43]. While previous cytotoxicity work has focused on *S. canadensis* QBAs, especially sanguinarine, our work adds to the limited evidence showing other constituents such as NDGA [44] and ZnCl_2,_ often the main constituents by weight in black salve, also possess skin cell cytotoxicity [45, 46].

Due to the significant cytotoxicity of sanguinarine the main alkaloid present in bloodroot rhizomes, low μM compound concentrations were used to check for the presence of synergistic compound cytotoxicity. We have shown for the first time that chelerythrine and minor QBAs at low concentrations not having a cytotoxic effect by themselves will boost the cytotoxic effects of sanguinarine. This suggests a synergistic rather than additive cytotoxic effect.

Sanguinarine as a lead drug compound has been found to possess multi-target pharmacology [14]. It acts as a DNA intercalator similar to the chemotherapy agent doxorubicin [47]; depletes nuclear topoisomerase II similar to the chemotherapy agent etoposide [48]; generates reactive oxygen species cytotoxicity [49]; disrupts G-quadruplex oncogenes such as KRAS [50] and c-Myc [51] and induces rapid cellular glutathione depletion [43]. Although similar in structure, the other alkaloids present in black salve seem to impact different molecular pathways perhaps explaining their potential for a synergistic cytotoxic effect. Chelerythrine for example, is a significant disruptor of Bcl-XL/ BH3 [52] and mammalian target of rapamycin (mTOR) [53], while sanguilutine activates receptor-interacting protein kinase 1 (RIP1) inducing programmed cell death by necroptosis [54].

In the A375 cell line this synergistic cytotoxicity seemed to disappear at a 3 μM concentration. One possible explanation for this loss of synergism being that at a 3 μM sanguinarine concentration, these pathways have been saturated such that additional alkaloids do not result in further cytotoxicity. In contrast in the A431 cell line, synergistic cytotoxicity largely became evident at a 3 μM alkaloid concentration. The A431 cell line appears to be more resistant to sanguinarine cytotoxicity than the A375 cell line, it may as a result require higher compound concentrations before synergistic cytotoxicity becomes evident.

The alkaloids present in black salve appear to have a more rapid cytotoxic onset of action compared to 5-FU, a topical skin cancer therapy currently in clinical use. This rapid cytotoxic action confirms previous cytotoxicity studies suggesting the application of black salve to human skin would result in rapid tissue necrosis. In a product, such as black salve, where the formulation is not standardized, quality assured or assessed by a therapeutic regulator, the chance of patient misadventure and harm is high. By the time a patient decides to remove the salve, significant damage may have already been done, including to the patient’s normal tissues.

Currently 5-FU when used to treat Bowens Disease, a type of squamous cell carcinoma confined to the epidermis, requires a prolonged treatment time of 6 weeks [55]. While topical therapies are not suggested for the treatment of non-superficial skin cancers [56]. Having a rapidly acting, powerful escharotic may sound clinically appealing as it could theoretically reduce treatment times improving patient compliance and broaden the scope of skin cancers that can be managed by topical therapies. The potential carcinogenicity of sanguinarine however [57] and the reported harm done to patients that have used black salve [58], suggest its use should move from the bedside back to the laboratory bench. The development of CAM treatments should not occur outside a rigorous safety and testing framework, the principle of “primum non nocere”, first do no harm is as relevant to CAM as it is to traditional pharmaceutical development.

Further toxicity work is urgently required to determine whether safe compound levels exist in multicomponent medicines like black salve. Due to the potential interference from fluorescent compounds and the alteration of their fluorescence in the presence of DNA, future fluorescent alkaloid cytotoxicity assay testing should be conducted on multiple cytotoxicity platforms. As herbal medicines become increasingly popular, developing flexible platforms able to simultaneously assess the cytotoxicity of individual and combination compounds would greatly aid in the toxicological assessment and therapeutic development of these natural products.

## Supplementary Information

**Additional file 1.**

## Data Availability

The datasets used/ and or analysed during the current study are available from the corresponding author on reasonable request.
