# Supplementary material for: Understanding of black salve toxicity by multi-compound cytotoxicity assays
Source: BMC Complement Med Ther. 2022 Sep 20;22:247. doi: 10.1186/s12906-022-03721-y (PMC9487053; doi:10.1186/s12906-022-03721-y)
Supplement: Supplementary file 1 — Additional file 1. [file 12906_2022_3721_MOESM1_ESM.pptx]

## Slide 1
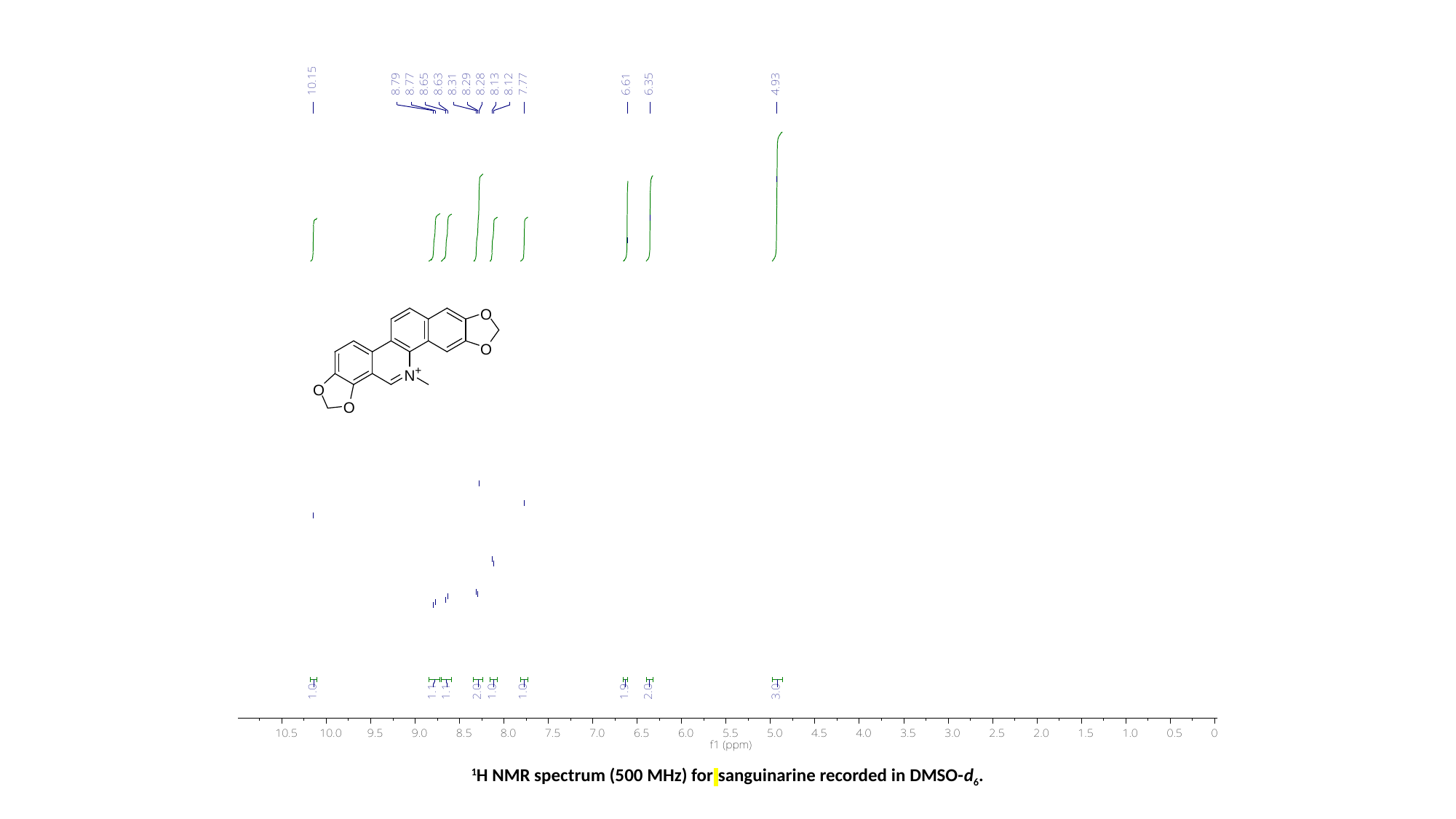

1H NMR spectrum (500 MHz) for sanguinarine recorded in DMSO-d6.

## Slide 2
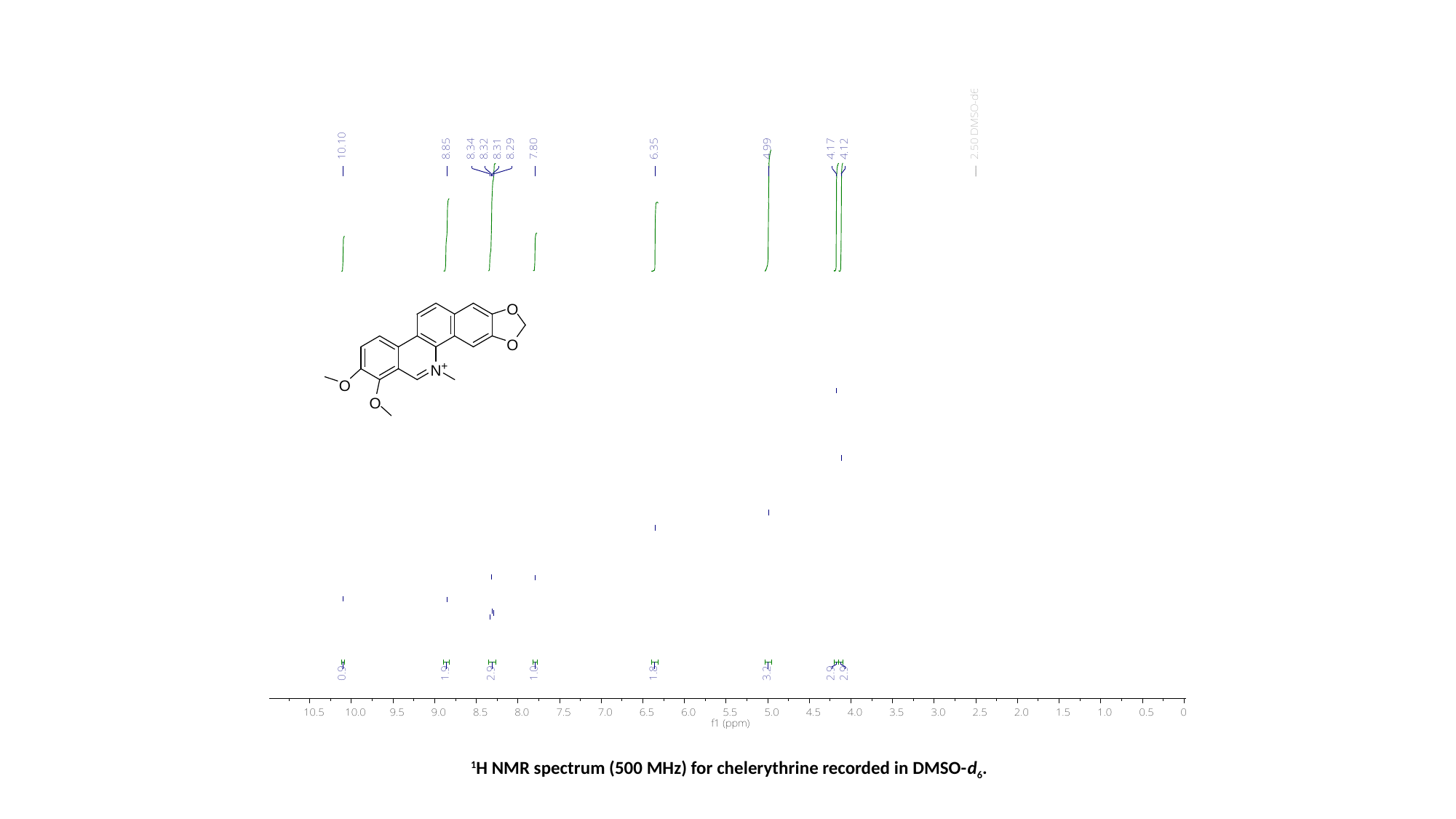

1H NMR spectrum (500 MHz) for chelerythrine recorded in DMSO-d6.

## Slide 3
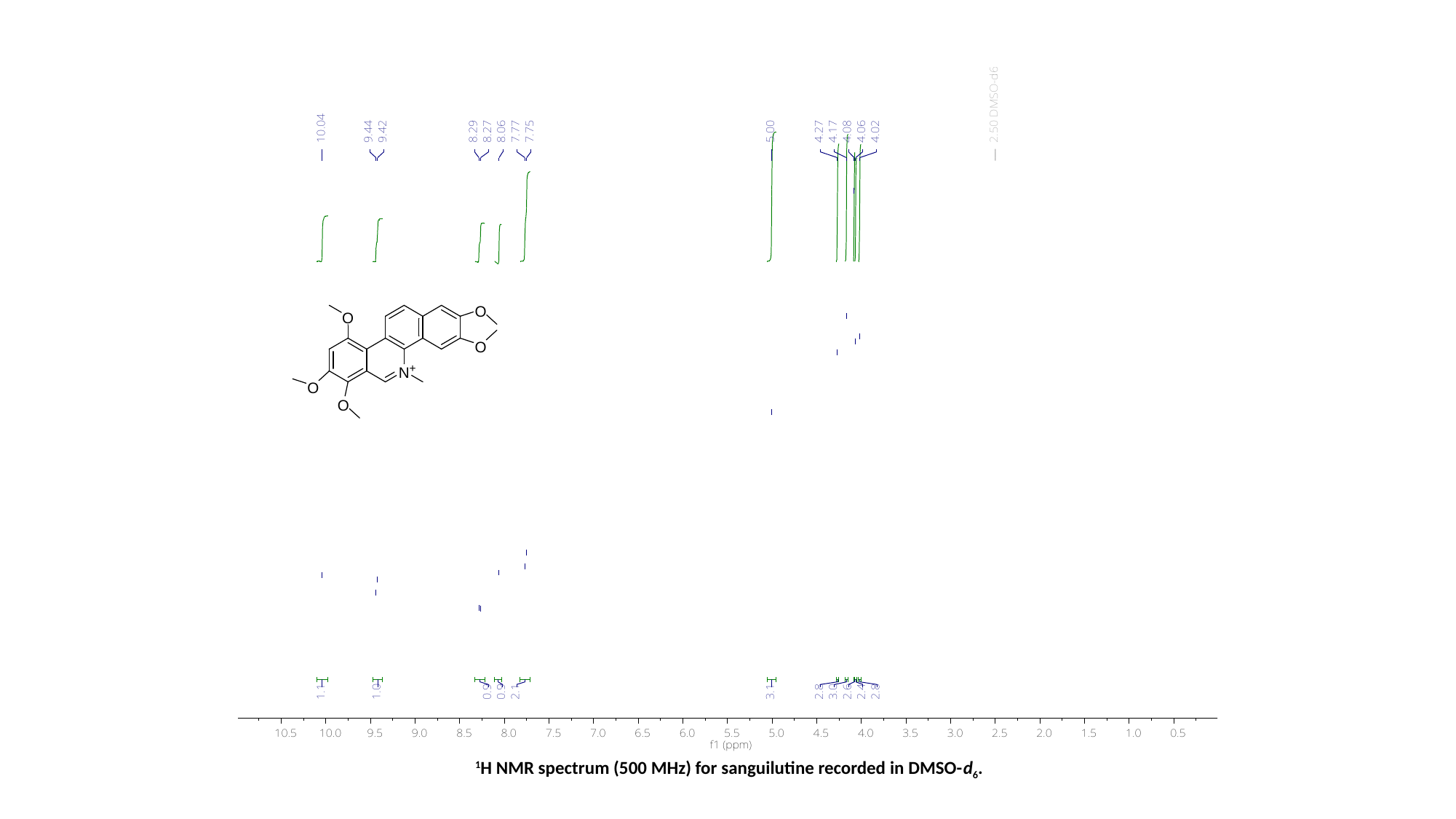

1H NMR spectrum (500 MHz) for sanguilutine recorded in DMSO-d6.

## Slide 4
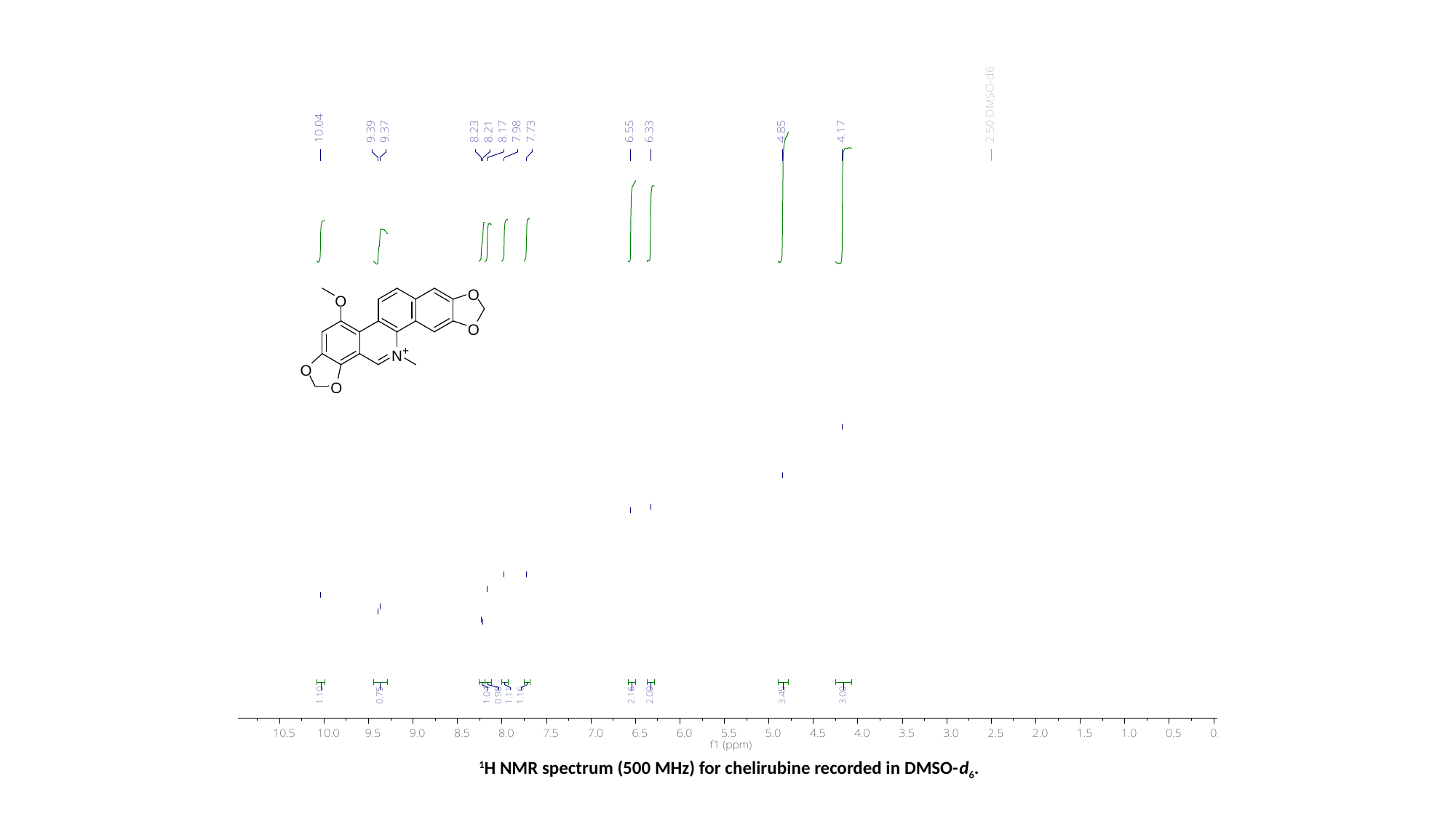

1H NMR spectrum (500 MHz) for chelirubine recorded in DMSO-d6.

## Slide 5
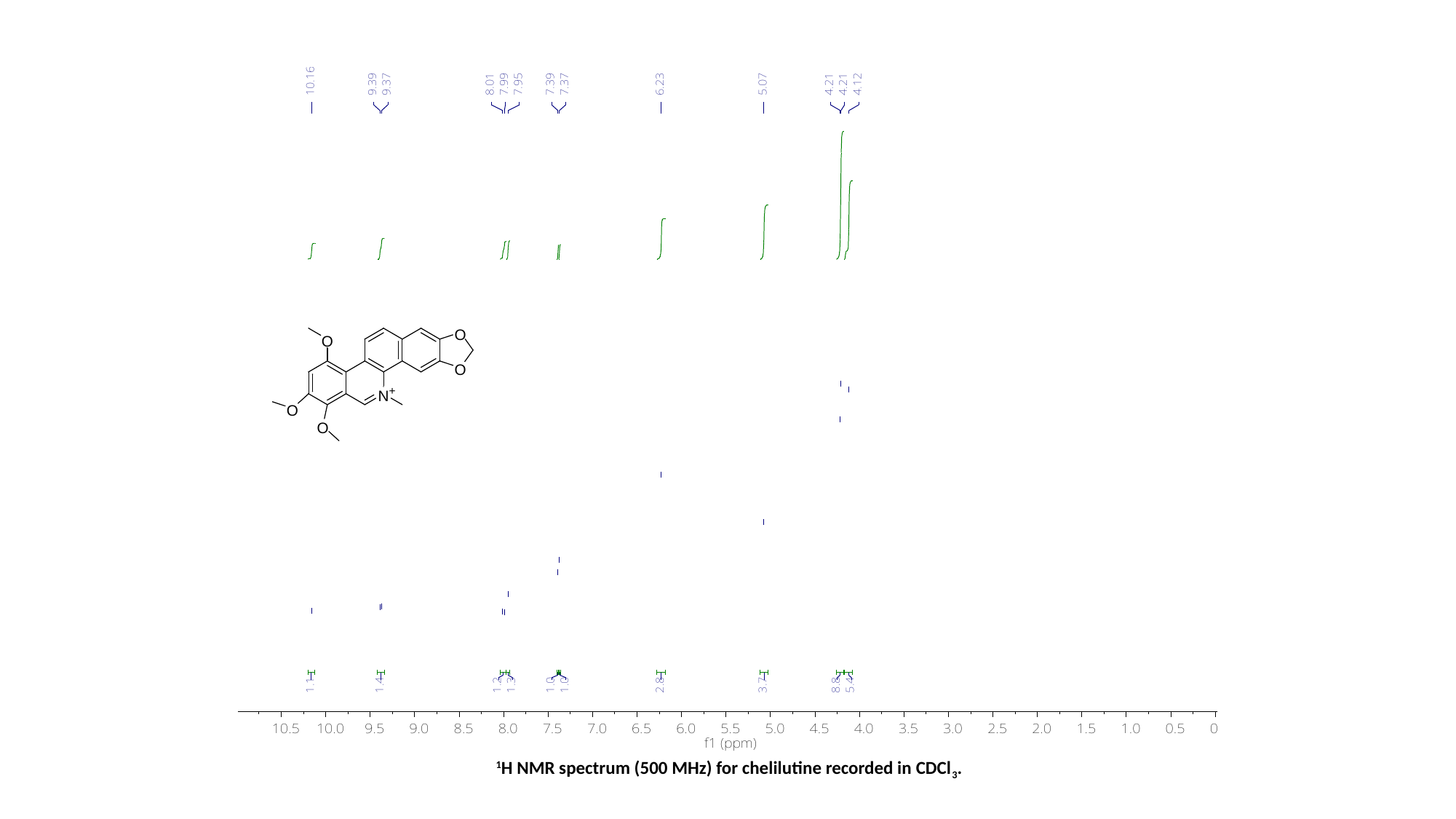

1H NMR spectrum (500 MHz) for chelilutine recorded in CDCl3.

## Slide 6
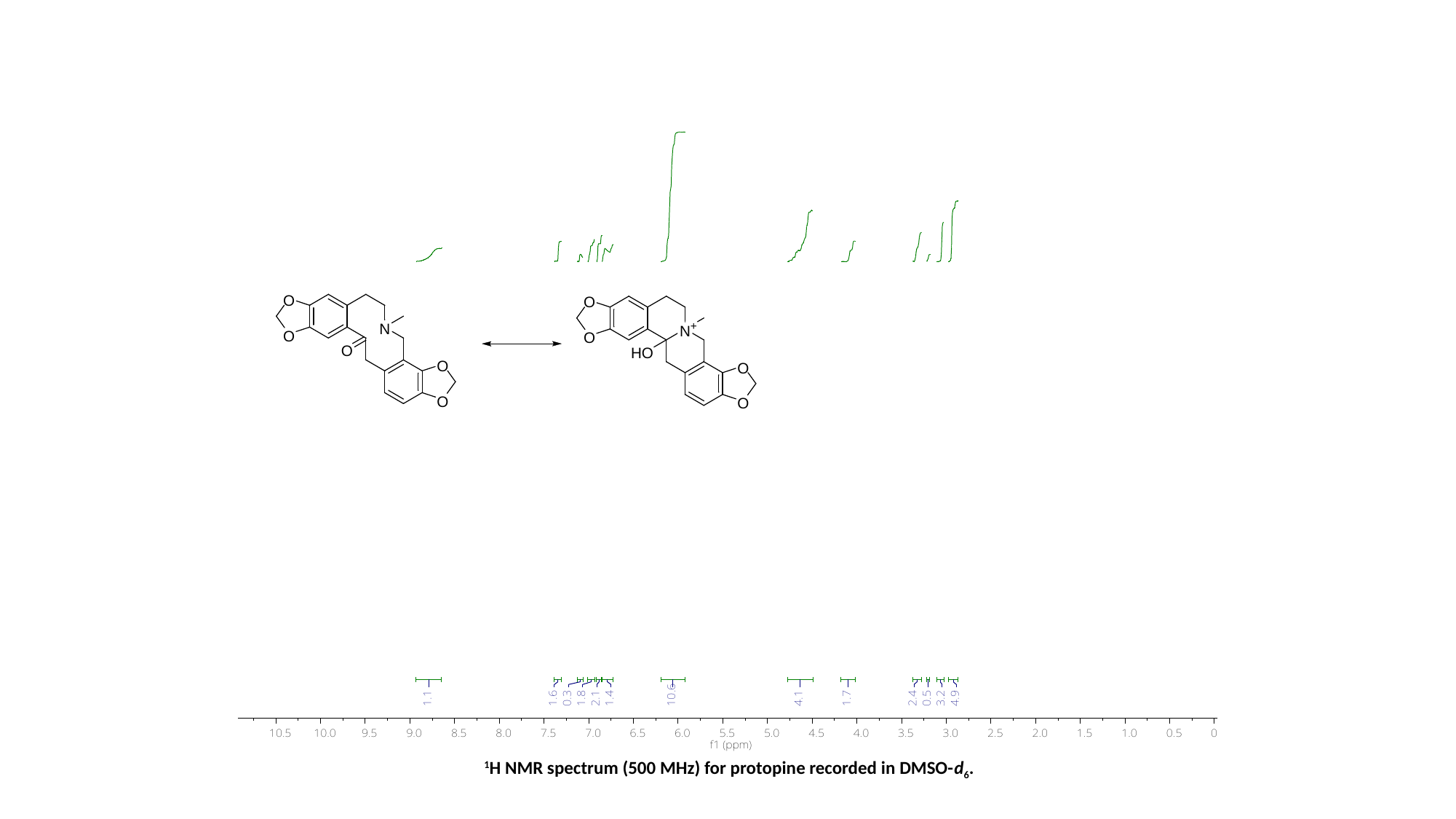

1H NMR spectrum (500 MHz) for protopine recorded in DMSO-d6.

## Slide 7
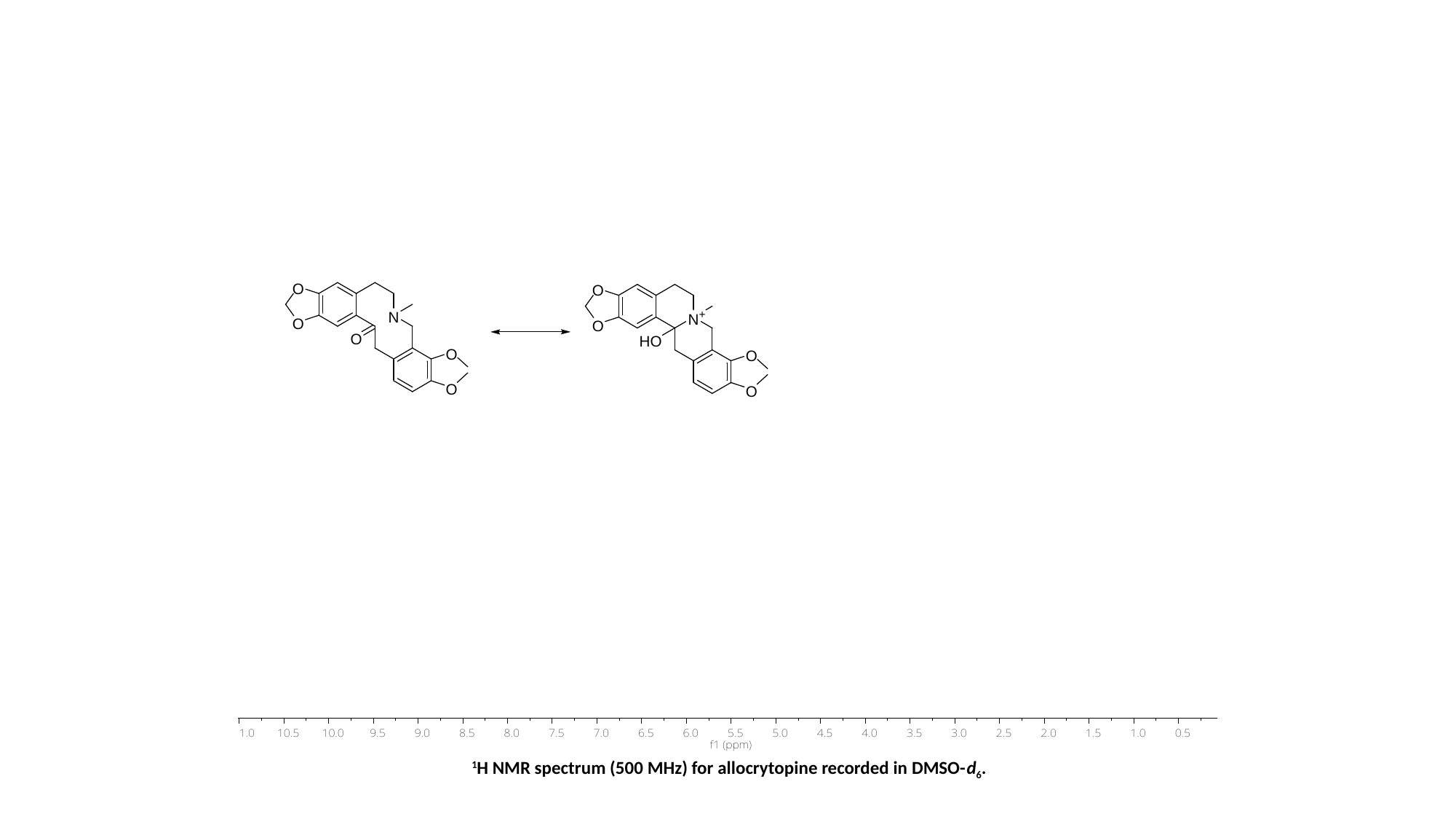

1H NMR spectrum (500 MHz) for allocrytopine recorded in DMSO-d6.
